# Whole-Genome DNA Methylation Profile of the Jewel Wasp (*Nasonia vitripennis*)

**DOI:** 10.1534/g3.113.008953

**Published:** 2013-12-30

**Authors:** Suzannah M. Beeler, Garrett T. Wong, Jennifer M. Zheng, Eliot C. Bush, Emily J. Remnant, Benjamin P. Oldroyd, Robert A. Drewell

**Affiliations:** * Biology Department, 301 Platt Boulevard, Harvey Mudd College, Claremont, California 91711; † Behaviour and Genetics of Social Insects Laboratory, School of Biological Sciences A12, University of Sydney, New South Wales 2006, Australia; ‡ Department of Biological Sciences, Mount Holyoke College, South Hadley, Massachusetts 01705; § Department of Biology, Amherst College, Amherst, Massachusetts 01002

**Keywords:** DNA methylation, *Nasonia*, epigenetics

## Abstract

The epigenetic mark of DNA methylation, the addition of a methyl (CH_3_) group to a cytosine residue, has been extensively studied in many mammalian genomes and, although it is commonly found at the promoter regions of genes, it is also involved in a number of different biological functions. In other complex animals, such as social insects, DNA methylation has been determined to be involved in caste differentiation and to occur primarily in gene bodies. The role of methylation in nonsocial insects, however, has not yet been explored thoroughly. Here, we present the whole-genome DNA methylation profile of the nonsocial hymenopteran, the jewel wasp (*Nasonia vitripennis*). From high-throughput sequencing of bisulfite-converted gDNA extracted from male *Nasonia* thoraces, we were able to determine which cytosine residues are methylated in the entire genome. We found that an overwhelming majority of methylated sites (99.7%) occur at cytosines followed by a guanine in the 3′ direction (CpG sites). Additionally, we found that a majority of methylation in *Nasonia* occurs within exonic regions of the genome (more than 62%). Overall, methylation is sparse in *Nasonia*, occurring only at 0.18% of all sites and at 0.63% of CpGs. Our analysis of the *Nasonia* methylome revealed that in contrast to the methylation profile typically seen in mammals, methylation is sparse and is constrained primarily to exons. This methylation profile is more similar to that of the social hymenopteran species, the honey bee (*Apis mellifera*). In presenting the *Nasonia* methylome, we hope to promote future investigation of the regulatory function of DNA methylation in both social and nonsocial hymenoptera.

DNA methylation is the process by which methyl (CH_3_) groups are added to cytosine residues in genomic DNA (Li 2002). The addition of a methyl group occurs predominately at cytosines that are followed by a guanine in the 3′ direction (known as CpG sites) and allows the genome to hold more information than the sequence of the four bases alone. Thus, methylation patterns are of great interest. DNA methylation is particularly prevalent in mammals, in which 60%–90% of CpGs are methylated across the entire genome (Lister *et al.* 2009) and methylation is found at both promoters and intergenic regions (Elango and Yi 2008). In mammals, DNA methylation has several well-characterized regulatory functions, including X chromosome inactivation, genomic imprinting, alternative splicing, and cellular differentiation (Schwartz and Ast 2010). However, the role of methylation in other complex animals, including insects, is not as well-understood (Drewell *et al.* 2012).

In insects, patterns of DNA methylation differ strongly from those observed in mammals (Glastad *et al.* 2011; Lyko and Maleszka 2011), suggesting that the regulatory function of methylation within these organisms also differs (Drewell *et al.* 2012; Patalano *et al.* 2012). First, some insect species, such as *Drosophila melanogaster*, have unmethylated genomes and lack both the maintenance (Dnmt1) and *de novo* (Dnmt3) DNA methyltransferases (Lyko and Maleszka 2011). Second, those insects that have the enzymatic machinery to perform DNA methylation, such as the honey bee *Apis mellifera*, show much lower overall levels of methylation (approximately 0.69% of all CpGs are methylated) than mammals, and methylation occurs primarily in exonic regions (Glastad *et al.* 2011; Lyko *et al.* 2010). In recent years, analyses of a number of additional insect methylomes have been performed, including those of the silkworm (Xiang *et al.* 2010), fire ant (Wurm *et al.* 2011), and desert locust (Falckenhayn *et al.* 2013). The role of DNA methylation in insects is particularly interesting because its functional activity is not entirely conserved across all the different species analyzed to date (Flores and Amdam 2011). The majority of functional studies of DNA methylation in Hymenoptera have focused on the eusocial honey bee *Apis mellifera* and it is now clear that methylation is central to caste polyphenism (Herb *et al.* 2012; Li-Byarlay *et al.* 2013). For example, more than 550 genes show different patterns of methylation between queens and workers (Lyko *et al.* 2010; Shi *et al.* 2012). The functional role of DNA methylation in nonsocial hymenoptera is especially intriguing because methylation does not play a role in caste differentiation, as is the case in their social relatives.

The parasitic jewel wasp, *Nasonia vitripennis*, provides a suitable species for studying the function of DNA methylation in nonsocial hymenoptera because its genome has recently been sequenced (Werren *et al.* 2010). One significant discovery from this sequencing project was that *Nasonia* has both Dnmt1 and Dnmt3 (Park *et al.* 2011), the two enzymes that are essential for DNA methylation. As an emerging model species, *Nasonia* therefore potentially represents a new methylome for studies aimed at generating a better understanding of the functional role of methylation in hymenopterans. Some functions of DNA methylation within *Nasonia* are already understood. DNA methylation is essential for development in *Nasonia*, because a knockdown of maternally provided *Dmnt1a* is lethal to embryos (Zwier *et al.* 2012). DNA methylation is involved in alternative splicing of genes related to sex determination (Park *et al.* 2011). Park *et al.* (2011) found for the small number of genes they examined, DNA methylation is generally sparse, occurring primarily within exonic regions, a result that is consistent with the honey bee methylome (Lyko *et al.* 2010). Here, we present the whole-genome DNA methylation profile of *Nasonia vitripennis*. Our goal was to provide an additional methylome to those already available from insects, so that the role of methylation in social and nonsocial Hymenoptera may be better-understood in future comparative studies.

## Materials and Methods

### DNA sources

*Nasonia vitripennis* were reared under standard laboratory conditions (Werren and Loehlin 2009) and the thoraces from 150 adult males were collected. Extraction of genomic DNA was performed using the DNeasy Blood and Tissue Kit (Qiagen) following the protocol for purification of total DNA from insects. A total of 10.7 µg gDNA was obtained. As a control for bisulfite conversion, 107 ng lambda phage DNA (N3011S; New England Biolabs) purified with PureLing Quick PCR Purification Kit (Invitrogen) was added to the sample.

### Sequencing of bisulfite-converted DNA libraries

Library construction, bisulfite conversion, and sequencing were performed at the Beijing Genomics Institute. Briefly, DNA was fragmented into 100-bp to 300-bp fragments by sonication (Covaris S-2; Woburn USA). The fragmentation parameters were as follows: duty cycle, 10%; intensity, 5; cycles/burst, 200; cycles, 16; and total fragmentation time, 960 sec. Fragmentation was confirmed using a 2100 Bioanalyzer (Aligent Technologies). Fragments were end-repaired (Illumina) as recommended by the manufacturer. Repaired fragments were ligated with methylated sequencing adaptors using a paired end adaptor oligo kit and oligo mix 5 (Illumina). Ligated fragments were selected by gel electrophoresis and fragments of 360 bp were extracted using a QIAquick gel extraction kit (Qiagen).

Size-selected fragments were bisulfite-treated using an EZ-DNA methylation kit (Zymo Research) and enriched using a MethyMiner methylated DNA enrichment kit (Invitrogen). It should be noted that this kit uses the DNA binding domain from human methyl-binding domain 2 protein to enrich for methylated DNA; therefore, when compared to experimental approaches that do not use this step, it will likely introduce selection for methylated fragments. Libraries were amplified using T4 polymerase (Enzymatics) and sequenced on the Illumina HiSeq platform.

### Sequence analysis and mapping DNA methylation

Data were filtered to remove adaptor sequences, duplicate sequences, contamination, and low-quality reads using BGI software. For methylation analysis, we followed the methods of Lyko *et al.* (2010). We mapped our reads onto the *Nasonia vitripennis* genome assembly 1.0 (Munoz-Torres *et al.* 2011) using BSMAP version 2.6 (Xi and Li 2009) with seed size of 12 and maximum allowed mismatches of 5. Similarly, we mapped our reads onto the complete lambda phage genome (GenBank: J02459.1); 73% of our reads mapped onto the Nasonia genome and 22% mapped onto the lambda genome. We considered only reads that mapped uniquely, bases within reads that had a quality score of 20 or more, and those that were next to three matches with quality scores of at least 15 (Altshuler *et al.* 2000). From these data we determined the number of converted and unconverted reads at each C position in the *Nasonia* and lambda genomic assemblies, accounting for the fact that each read came from a bisulfite reaction on one strand or the other.

To estimate the overall rate of bisulfite conversion in nonmethylated bases in our experiments, we used the C-to-T conversion rate in the lambda phage DNA, in which all cytosines should have been converted. We found that 99.28% of cytosines were converted in the lambda DNA. We also determined the conversion rate by examining the rate of C-to-T conversion at cytosines that were not in a CpG context because we had found these sites to be virtually unmethylated in *Nasonia* (<0.3%). For our data, 99.71% of these were converted to T. Both methods of determining the bisulfite conversion rate indicated that our false-negative rate was less than 1%.

To identify individual cytosines that were significantly methylated in the *Nasonia* genome, we compared the number of converted and nonconverted reads at each site. We used only sites that had coverage of more than 1 and less than 31 reads. We asked how likely these counts were under a binomial distribution in which the probability of success is one minus the conversion rate, and we corrected this probability value for multiple testing (Benjamini and Hochberg 1995). From this, we were able to determine the top methylated genes by the number of methylated sites and by the proportion of methylated sites.

## Results and Discussion

**Figure 1:**
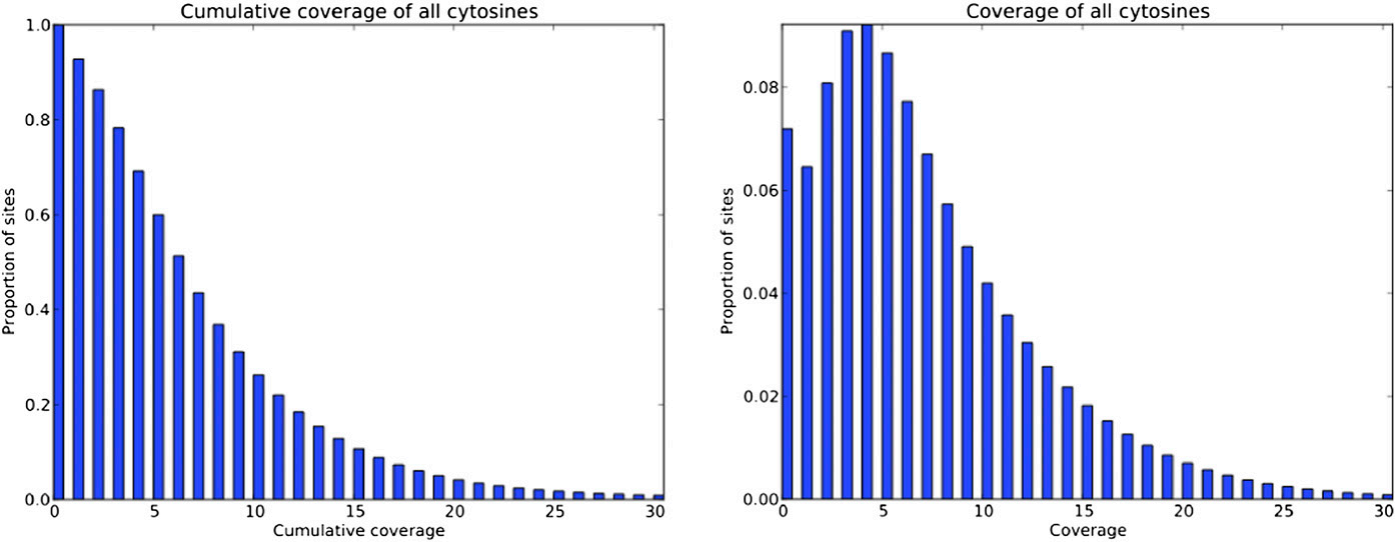
Sequencing coverage over all cytosines in male *Nasonia* thorax. (Left) Cumulative coverage, *e.g.*, approximately 20% of sites are covered by 11 or more reads. (Right) The proportion of sites that have a certain level of coverage, *e.g.*, approximately 2% of sites have coverage of 14 reads. Note that every read can trace its origin to a bisulfite conversion event that happened on one strand or the other. Here, we only counted reads that were on the correct strand to be informative at a particular cytosine.

Sequencing of bisulfite-converted DNA from male *Nasonia vitripennis* thoraces (along with whole lambda bacteriophage DNA as a control) allowed us to obtain a dataset of 65 million reads after quality control (see *Materials and Methods* for full details). To estimate the overall rate of bisulfite conversion in nonmethylated bases in our experiments, we measured the C-to-U deamination rate in the unmethylated bacteriophage lambda DNA as a spike in control. We found that 99.28% of cytosines were converted in the lambda DNA (see *Materials and Methods*), indicating that the false-negative rate was less than 1%. Median coverage of CpG sites in the *Nasonia* genome assembly was eight reads, with 86.4% of sites covered by two or more reads (Figure 1). The complete dataset of sequence reads is available at drewell.sites.hmc.edu/Nasonia_methylome.

Overall, a relatively small proportion of cytosines are methylated in the *Nasonia* genome, approximately 176,000 sites, or 0.18% of all sites and 0.63% of all CpGs. Methylation occurs predominately at CpG dinucleotides, accounting for 99.7% of methylated sites (Table 1). At 68,071 individual CpGs, methylation occurs on both DNA strands, accounting for 136,142 (77.4%) of all the methylated CpGs in the genome. The majority of methylation occurs within exonic regions, accounting for more than 62% of all methylated sites (Table 2). Among these significantly methylated sites there are many examples of individual sites that are methylated in 100% of the reads, although in some cases this may be a reflection of the overall low number of reads at some of these sites. In addition, we could not detect any evidence of significant methylation at the annotated transposable elements in the *Nasonia* genome (Werren *et al.* 2010). This methylome profile mirrors previous observations in the adult honey bee methylome (Lyko *et al.* 2010) and in the small subset of *Nasonia* genes previously analyzed (Park *et al.* 2011), indicating that DNA methylation outside of gene bodies and at non-CpG residues is rare within Hymenoptera. These results are drastically different from the methylation profile seen in mammals, in which 60%–90% of CpGs are methylated across the entire genome (Lister *et al.* 2009) and widespread methylation at transposons is involved in transcriptional suppression (Jones and Takai 2001).

**Table 1:** Methylated cytosines in CG, CHG, and CHH genomic contexts (H = A, T, or C)

Cytosines	Sites in Genome	Methylated Sites	% of All mCs
CG	28,048,814	175,884	99.7
CHG	16,637,411	97	0.055
CHH	54,832,489	431	0.244
Unclassified	19,320	1	5.67 × 10^−4^
Total	99,538,034	176,413	

Methylation occurs predominantly as CG sites.

**Table 2:** Mapping DNA methylation to genomic regions

Genomic Location	CGs	mCGs	% mCGs	% of All mCG
Exons	2,625,400	109,496	4.17	62.07
Introns	8,935,722	31,386	0.351	17.79
Intergenic regions	16,487,692	35,531	0.216	20.14

The values represent the percentage of CGs that are methylated within a particular genomic region and the percentage of all methylation sites of a given type that are in a particular region. For example, 4.17% of CGs are methylated in exons and 62.07% of all mCpGs are in exons.

**Figure 2:**
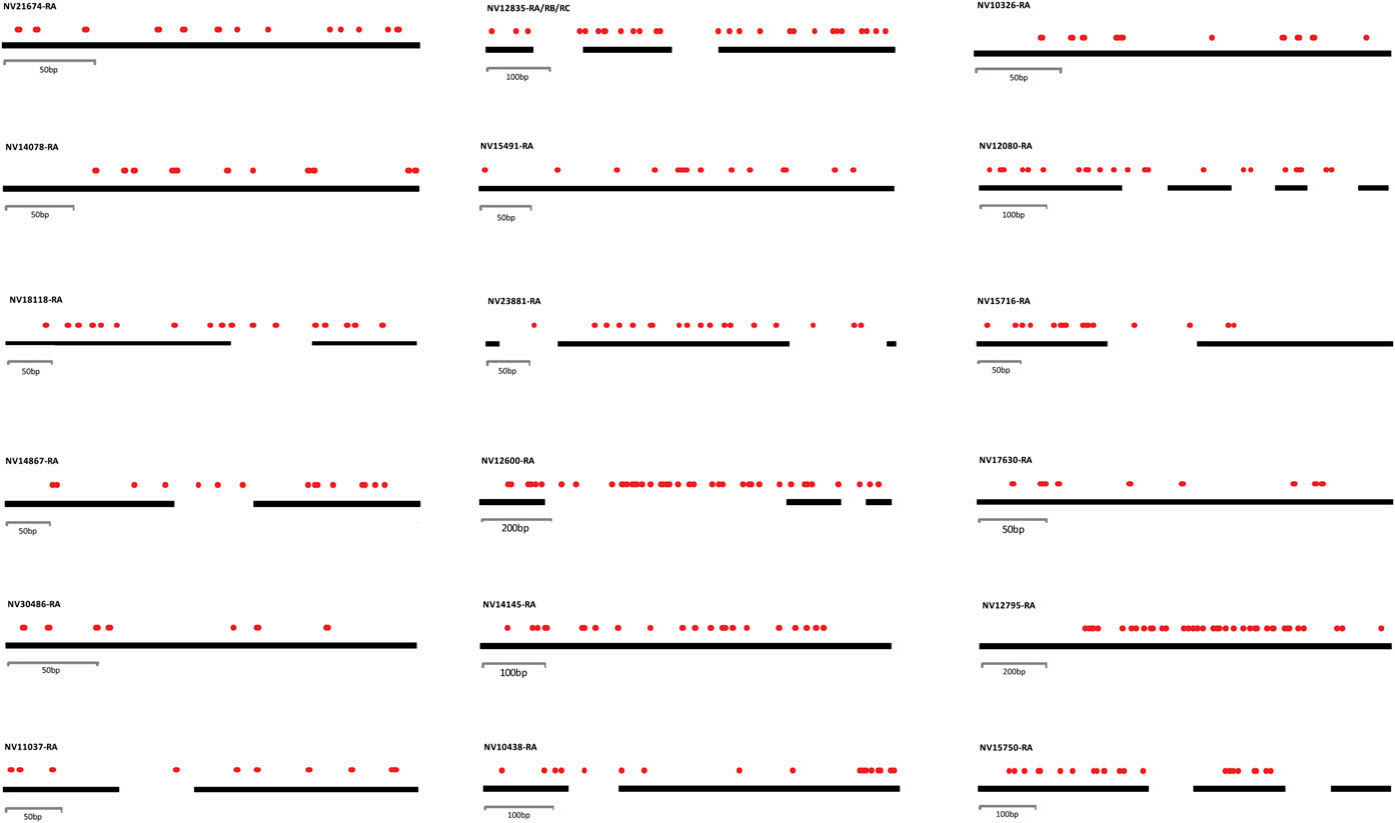
Annotations of methylation patterns for the top 20 most methylated genes by proportion. Black bars represent exonic regions and red circles represent methylated sites. Genes are presented in order of ranking from left to right and then top to bottom. The three isoforms of NV12835 are presented as a single annotation.

In an effort to investigate the highly methylated genes in the *Nasonia* genome, we analyzed the 20 most methylated genes by the proportion (number of methylated sites over number of base pairs in each annotated gene) (Table 3) and by the total number of methylated sites (Supporting Information, Table S1). It is important to note that determining the most methylated genes by proportion is potentially biased toward shorter genes, whereas determining most methylated genes by number of sites is biased toward longer genes. Eight of the top methylated genes by proportion consist of only a single exon, lacking any intronic regions. All but one of the genes with more than one exon have at least one mCpG in an intron (Table 3 and Figure 2). In general, the most methylated genes mirror the overall methylation profile of the genome, where a majority of methylation occurs in exonic regions. The 13^th^ ranked NV12600-RA is the only gene to deviate from this pattern, showing extensive methylation within its first intronic region (Figure 2). Additionally, in a number of the most frequently methylated genes (including NV12600-RA), there are methylated sites in close proximity to the exon–intron boundaries (Figure 2), a pattern that may be consistent with a possible functional role for methylation in regulating alternative splicing (Flores *et al.* 2012). The overall pattern of high levels of methylation in exons in *Nasonia* is consistent with a potential role in the regulation of splicing, as has been previously found in honeybees (Flores *et al.* 2012), where exons that are included during splicing often have higher levels of methylation at the start and at the end of the exon when compared to skipped exons.

**Table 3:** List of top 20 methylated genes by proportion of gene

Gene Name	Scaffold Number	Proportion Methylated	Sites Methylated	% of Sites in Introns
NV21674-RA	1	0.08811	20	NA
NV12835-RC*^a^*	9	0.08025	52	9.26
NV12835-RB*^a^*	9	0.08025	52	9.26
NV12835-RA*^a^*	9	0.08025	52	9.26
NV10326-RA	1	0.07347	18	NA
NV14078-RA	16	0.07023	21	NA
NV15491-RA	27	0.06983	28	NA
NV12080-RA	7	0.06852	42	28.6
NV18118-RA	136	0.06739	31	19.4
NV23881-RA	1270	0.06485	31	19.4
NV15716-RA	28	0.06237	30	13.3
NV14867-RA	21	0.06080	29	17.2
NV12600-RA	9	0.05908	68	73.5
NV17630-RA	94	0.05902	18	NA
NV30486-RA	18	0.05727	13	NA
NV14145-RA	16	0.05719	37	NA
NV12795-RA	9	0.05534	70	NA
NV11037-RA	3	0.05495	20	10.0
NV10438-RA	1	0.05491	33	3.03
NV15750-RA	29	0.05487	40	0.00

The percentages of methylated sites that occur in intronic regions are provided for genes that have more than one exon.

a Three genes marked with an asterisk are isoforms of the same gene.

**Figure 3:**
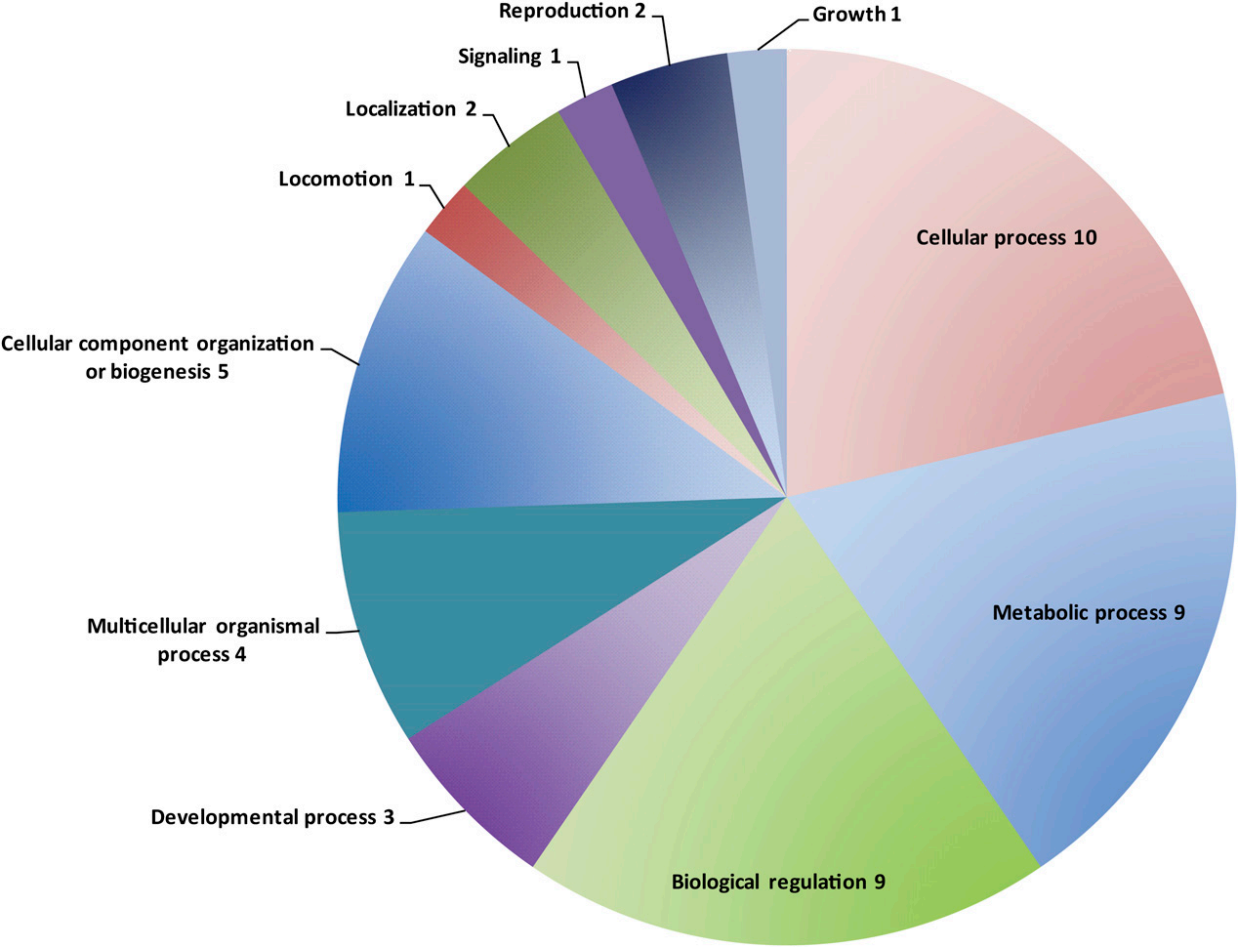
Gene Ontology (GO) categories associated with the top 20 methylated genes by proportion of gene.

We used the predicted protein sequence of the 20 most methylated genes from NasoniaBase (Munoz-Torres *et al.* 2011) (by proportion and by number of sites) to assign Gene Ontology (GO) terms in Blast2Go based on homology to the top BLAST hit and the corresponding GO annotations (Götz *et al.* 2008). GO analysis revealed an enrichment of genes involved in metabolic and cellular processes and biological regulation in the top methylated genes by proportion (Figure 3 and Table S2). Intriguingly, the seventh ranked gene by proportion, NV15491-RA, is a putative methyltransferase (Table S2). The top methylated genes by number of sites were also enriched for cellular and metabolic processes (Figure S1 and Table S3). Analysis of the top 1% of methylated genes in the genome, by either proportion or total number of sites, revealed similar GO term distributions to the top 20 genes (data not shown).

## Conclusions

Many aspects of the DNA methylation profile of *Nasonia vitripennis* are similar to the patterns of methylation in the social relative of *Nasonia*, the honey bee. First, methylation occurs overwhelmingly only at CpG sites (99.7% of sites). Additionally, although a majority of the methylation appears on promoters within mammalian methylomes, *Nasonia* primarily has methylation within gene bodies. Overall, methylation within the *Nasonia* genome is sparse (0.18% of all sites and 0.63% of CpGs). The GO terms for the most methylated genes in *Nasonia* indicate enrichment of genes involved in cellular and metabolic processes and biological regulation. Given the recent indication that DNA methylation patterns in insects can be tissue-type–specific and/or cell-type–specific (Foret *et al.* 2012), it is important to note that the methylome in our study is from a heterogeneous mix of cells in the wasp thorax. However, the *Nasonia* methylome can be used for comparative studies of methylomes within the order Hymenoptera. Previous studies of just a few genes determined that epigenetic marks, specifically DNA methylation, could play a role in alternative splicing of genes involved in sex determination (Park *et al.* 2011; Verhulst *et al.* 2010). Several aspects of the DNA methylation profile of the *Nasonia* genome are consistent with DNA methylation having a role in the regulation of alternative splicing (Flores *et al.* 2012). For example, a number of the top methylated genes have methylation in exons and/or are associated with exon–intron boundaries. By presenting the whole *Nasonia* methylome, we hope to catalyze further analysis of the regulatory function of DNA methylation in *Nasonia* specifically and Hymenoptera in general.

## Supplementary Material

Supporting Information
