# Supplementary material for: Whole-Genome DNA Methylation Profile of the Jewel Wasp (Nasonia vitripennis)
Source: G3 (Bethesda). 2013 Dec 30;4(3):383–8. doi: 10.1534/g3.113.008953 (PMC3962478; doi:10.1534/g3.113.008953)
Supplement: Supporting Information [file supp_4_3_383__index.html]

Whole-Genome DNA Methylation Profile of the Jewel Wasp (Nasonia vitripennis) — Supporting Information 

# Whole-Genome DNA Methylation Profile of the Jewel Wasp (*Nasonia vitripennis*)

## Supporting Information for Beeler *et al.*, 2014

**Files in this Data Supplement:**

- Supporting Information - Figure S1 and Tables S1-S3 (PDF, 279 KB)
- Figure S1 - Gene Ontology (GO) categories associated with top 20 methylated genes by number of sites. (PDF, 219 KB)
- Table S1 - List of top 20 methylated genes by number of sites. (PDF, 54 KB)
- Table S2 - Gene Ontology of top 20 methylated genes by proportion of gene (.xlsx, 13 KB)
- Table S3 - Gene Ontology of top 20 methylated genes by number of sites (.xlsx, 14 KB)
