# Supplementary material for: Whole-Genome DNA Methylation Profile of the Jewel Wasp (Nasonia vitripennis)
Source: G3 (Bethesda). 2013 Dec 30;4(3):383–8. doi: 10.1534/g3.113.008953 (PMC3962478; doi:10.1534/g3.113.008953)
Supplement: Supporting Information [file supp_g3.113.008953_008953SI.pdf]

G3 Genes | Genomes | Genetics

Whole genome DNA methylation profile of the jewel wasp (*Nasonia vitripennis*)

Suzannah M. Beeler[1], Garrett T. Wong[1], Jennifer M. Zheng[1], Eliot C. Bush[1], Emily J. Remnant[2], Benjamin P. Oldroyd[2] and Robert A. Drewell[1, 3, 4*]

[1] Biology Department
301 Platt Boulevard
Harvey Mudd College
Claremont, CA 91711, USA

[2] Behaviour and Genetics of Social Insects Laboratory
School of Biological Sciences A12
University of Sydney NSW 2006, Australia

[3] Department of Biological Sciences
Mount Holyoke College
South Hadley, MA 01705, USA

[4] Department of Biology
Amherst College
Amherst, MA 01002, USA

[*] To whom correspondence should be addressed: rdrewell@amherst.edu

DOI: 10.1534/g3.113.008953

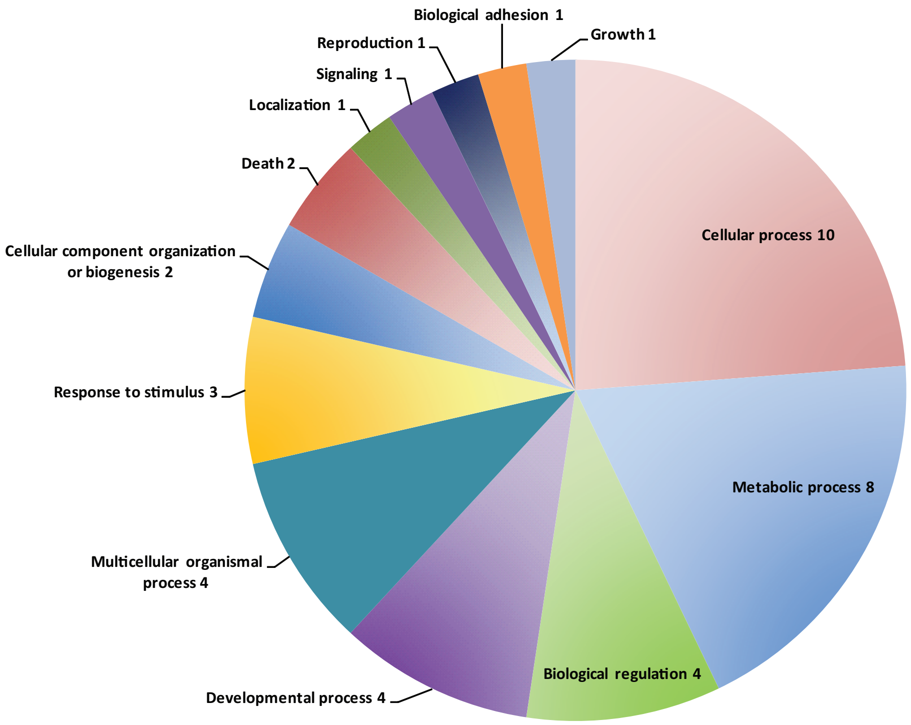

Figure S1 Gene Ontology (GO) categories associated with top 20 methylated genes by number of sites.

Table S1 List of top 20 methylated genes by number of sites.

Gene Name	Scaffold Number	Sites Methylated	Proportion Methylated
NV24953-RA	170	290	0.001619
NV22871-RA	29	186	0.002679
NV18125-RA	136	153	0.01406
NV11634-RA	5	152	0.009967
NV11765-RA	5	144	0.01835
NV17924-RA	123	142	0.02147
NV20551-RA	369	128	0.008775
NV21944-RA	173	128	0.001552
NV17591-RA	92	127	0.004106
NV12649-RA	9	127	0.01232
NV16482-RA	40	126	0.01306
NV20764-RA	7	125	0.02251
NV18726-RA	357	125	0.01268
NV10210-RA	1	125	0.007337
NV18909-RA	595	123	0.008764
NV23174-RA	95	122	0.0008480
NV13733-RA	15	122	0.01218
NV25328-RA	108	122	0.001963
NV18787-RA	417	121	0.005422
NV15194-RA	23	120	0.03221

Tables S2-S3 are available for download as Excel files at http://www.g3journal.org/lookup/suppl/doi:10.1534/g3.113.008953/-/DC1.

Table S2 Gene Ontology of top 20 methylated genes by proportion of gene. Sequence description and Gene Ontology (GO) terms were assigned by Blast2Go.

Table S3 Gene Ontology of top 20 methylated genes by number of sites. Sequence description and Gene Ontology (GO) terms were assigned by Blast2Go.
