# Supplementary figures and images for: Whole-Genome DNA Methylation Profile of the Jewel Wasp (Nasonia vitripennis)
Source: G3 (Bethesda). 2013 Dec 30;4(3):383–8. doi: 10.1534/g3.113.008953 (PMC3962478; doi:10.1534/g3.113.008953)

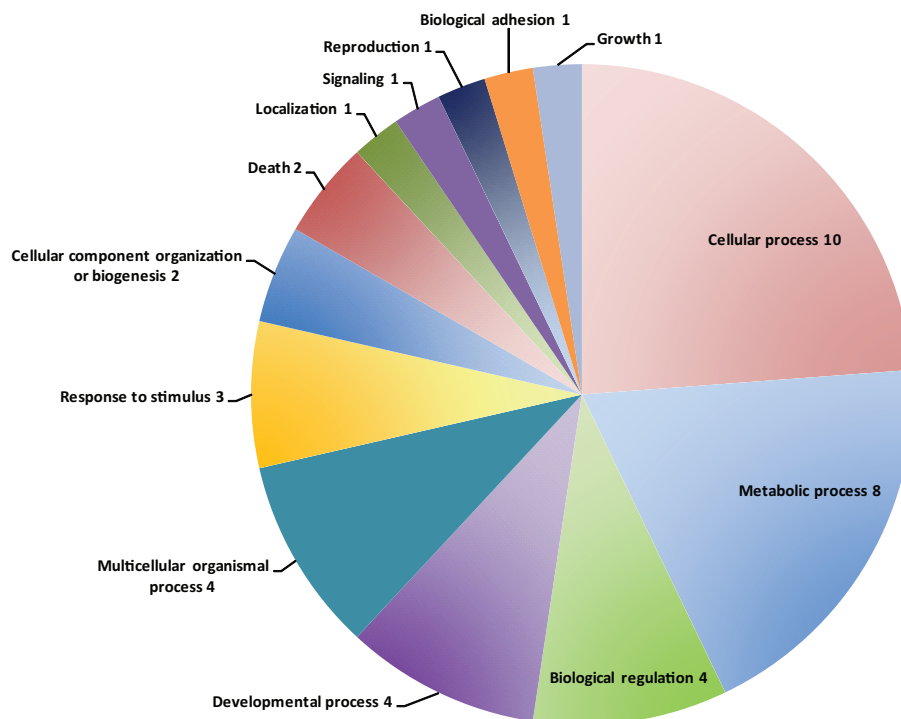

**Figure S1** Gene Ontology (GO) categories associated with top 20 methylated genes by number of sites.

Supplement: Supporting Information [file supp_g3.113.008953_FigureS1.pdf]
