# Supplementary material for: Whole-Genome DNA Methylation Profile of the Jewel Wasp (Nasonia vitripennis)
Source: G3 (Bethesda). 2013 Dec 30;4(3):383–8. doi: 10.1534/g3.113.008953 (PMC3962478; doi:10.1534/g3.113.008953)
Supplement: Supporting Information [file supp_g3.113.008953_TableS1.pdf]

Table S1 List of top 20 methylated genes by number of sites.

Gene Name	Scaffold Number	Sites Methylated	Proportion Methylated
NV24953-RA	170	290	0.001619
NV22871-RA	29	186	0.002679
NV18125-RA	136	153	0.01406
NV11634-RA	5	152	0.009967
NV11765-RA	5	144	0.01835
NV17924-RA	123	142	0.02147
NV20551-RA	369	128	0.008775
NV21944-RA	173	128	0.001552
NV17591-RA	92	127	0.004106
NV12649-RA	9	127	0.01232
NV16482-RA	40	126	0.01306
NV20764-RA	7	125	0.02251
NV18726-RA	357	125	0.01268
NV10210-RA	1	125	0.007337
NV18909-RA	595	123	0.008764
NV23174-RA	95	122	0.0008480
NV13733-RA	15	122	0.01218
NV25328-RA	108	122	0.001963
NV18787-RA	417	121	0.005422
NV15194-RA	23	120	0.03221
